# Retest effects in a diverse sample: sociodemographic predictors and possible correction approaches

**DOI:** 10.1590/1980-5764-DN-2021-0027

**Published:** 2022-04-29

**Authors:** Laiss Bertola, Isabela Judith Martins Benseñor, Andre Russowsky Brunoni, Paulo Caramelli, Sandhi Maria Barreto, Arlinda Barbosa Moreno, Rosane Harter Griep, Maria Carmen Viana, Paulo Andrade Lotufo, Claudia Kimie Suemoto

**Affiliations:** 1 Universidade de São Paulo, Faculdade de Medicina, São Paulo SP, Brazil.; 2 Universidade de São Paulo, Hospital Universitário, Centro de Pesquisa Epidemiológica e Clínica, São Paulo SP, Brazil.; 3 Universidade de São Paulo, Faculdade de Medicina, Departamento de Medicina Interna, São Paulo SP, Brazil.; 4 Universidade de São Paulo, Faculdade de Medicina, Departamento e Instituto de Psiquiatria, Laboratório de Neurociências, São Paulo SP, Brazil.; 5 Universidade Federal de Minas Gerais, Faculdade de Medicina, Departamento de Clínica Médica, Belo Horizonte MG, Brazil.; 6 Universidade Federal de Minas Gerais, Faculdade de Medicina, Hospital das Clínicas, Belo Horizonte MG, Brazil.; 7 Fundação Oswaldo Cruz, Escola Nacional de Saúde Pública Sérgio Arouca, Departamento de Epidemiologia e Métodos Quantitativos em Saúde, Rio de Janeiro RJ, Brazil.; 8 Fundação Oswaldo Cruz, Istituto Oswaldo Cruz, Laboratório de Educação em Saúde e Meio Ambiente, Rio de Janeiro RJ, Brazil.; 9 Universidade Federal do Espírito Santo, Departamento de Medicina Social, Vitória ES, Brazil.; 10 Universidade de São Paulo, Faculdade de Medicina, Divisão de Geriatria, São Paulo SP, Brazil.

**Keywords:** Reproducibility of Results, Aged, Longitudinal Studies, Psychometrics, Reprodutibilidade dos Testes, Idoso, Estudos Longitudinais, Psicometria

## Abstract

Repeated cognitive assessment in longitudinal studies favors the occurrence of retest effects, usually increasing the scores obtained at the follow-up assessments when compared to baseline. Therefore, retest effects can compromise the evaluation of cognitive decline in older adults.

**Objectives:**

We aimed to verify the occurrence of the retest effect and the impact of sociodemographic characteristics on the follow-up scores in a sample of 5,592 participants with a diverse sociodemographic profile, who were assessed twice during 4 years of follow-up.

**Methods:**

We tested two possible approaches to correct the retest effect and calculated the Reliable Change Index.

**Results:**

We observed increased scores at the follow-up assessment after 4 years, but the results indicate a modest occurrence of retest effects. The regression difference correction successfully generated follow-up corrected scores, while the mean difference did not provide effective corrections. Sociodemographic characteristics had a minor impact on the retest.

**Conclusions:**

We recommend the regression difference correction for retest effects. The absence of this methodological approach might lead to biased results using longitudinal cognitive scores.

## RESUMO.

Avaliações cognitivas repetidas em estudos longitudinais favorecem a ocorrência de efeitos de retestagem ou de prática, geralmente aumentando os escores obtidos nas avaliações de acompanhamento quando comparados aos da primeira avaliação. Sendo assim, os efeitos do retestagem podem comprometer a verificação do declínio cognitivo em idosos.

**Objetivos:**

Objetivamos verificar a ocorrência do efeito de prática e o impacto das características sociodemográficas nos escores de seguimento em uma amostra de 5.592 participantes com perfil sociodemográfico diverso, avaliada duas vezes durante quatro anos de seguimento.

**Métodos:**

Testamos duas abordagens possíveis para corrigir o efeito de prática e calculamos o índice de mudança confiável.

**Resultados:**

Observamos escores sutilmente maiores na avaliação de seguimento após quatro anos, o que sugere a ocorrência de efeitos de retestagem. A correção pela diferença da regressão gerou escores corrigidos de acompanhamento satisfatórios, enquanto a correção pela diferença média não forneceu correções eficazes. As características sociodemográficas tiveram impacto mínimo no efeito de prática.

**Conclusões:**

Recomendamos a forma de correção pela diferença da regressão para efeitos de retestagem. A ausência dessa abordagem metodológica, quando utilizamos escores cognitivos longitudinais, pode levar a resultados enviesados.

## INTRODUCTION

Longitudinal cognitive studies should consider the occurrence of practice or retest effects with repeated neuropsychological assessments. Repeated assessments with the same tests increase the occurrence of retest effects, usually increasing the score obtained at the follow-up assessment when compared to the first evaluation. Previous studies have shown that the second assessment shows the largest retest effects^1^. After three or more repeated cognitive assessments, there is a plateau in the retest effects^2,3^. Therefore, from the third assessment onward, the cognitive scores became more reliable due to the more stable retest effect^1,4,5^. The increase in the second assessment score might be due to several causes, including increased comfort in being tested, reduced anxiety at the follow-up visits for knowing what to expect, learning the test paradigm more than the items themselves, or even remembering test items. Besides, regression to the mean could be present since subjects with very low scores on the first assessment might increase their performance in subsequent evaluations^2,6^. These possible explanations can lead to increased cognitive scores at the second visit or they might even have caused slightly reduced performance at the first visit.

Retest effects produce unique repercussions in aging studies, compromising the expected observation of cognitive decline in older adults^7^. This phenomenon occurs because the average score gains in the presence of retest are often higher than the real cognitive change that happens during the follow-up period^2^.

It is also known that frequent assessments may obscure the real cognitive decline^5^ and that cognitive tests have distinct practice effects^1,8,9^. Previous studies have suggested the use of parallel tests to reduce the retest effects^7^. However, this solution depends on well-matched equivalent test forms to avoid measurement errors that can be erroneously interpreted as cognitive improvement or decline^10^.

Literature diverges about whether sociodemographic characteristics are related to the retest effects. Effects were reported to be higher in younger participants (18–53 years old compared to 54–97 years old)^4^, while other studies found that age and other sociodemographic variables (e.g., sex, education, and race/ethnicity) were not related to retest effects^8,11^. Although education was not previously related to retest effects, we hypothesized that individuals with low education are more prone to underperform in their first assessment due to unfamiliarity with testing situations.

Therefore, we assume that, if not considered in the analyses, retest effects can lead to biased cognitive results in longitudinal studies. Therefore, the aims of this study were to (1) verify the occurrence of retest effects in a longitudinal study, (2) verify whether sociodemographic characteristics are related to this effect, and (3) address how to take retest effects into account when using a data set with two visits.

## METHODS

### Participants

The ELSA-Brasil sample is composed of 15,105 active or retired employees from public institutions from six large Brazilian cities (e.g., Belo Horizonte, Porto Alegre, Rio de Janeiro, Salvador, São Paulo, and Vitória), of both sexes, aged between 35 and 74 years at baseline (2008–2010)^12,13^. The ELSA-Brasil is a longitudinal study investigating the incidence and evolution of chronic diseases, especially cardiovascular diseases and diabetes, among middle-aged and older adults. The exclusion criteria of this study were the presence of clinically observed severe cognitive or communication impairment, intention to quit work at the institution shortly for reasons not related to retirement, and, if retired, living outside the corresponding metropolitan area. Women currently or recently pregnant were rescheduled so that the first interview could take place at least 4 months after delivery. All participants were Brazilian-Portuguese speakers.

The baseline assessment in the study included sociodemographic information, clinical history, cognitive and mental health evaluation, lifestyle factors, occupational history, and family history of major diseases. Cognitive function was reassessed only in participants aged 55 years or older (7,066 eligible participants) at the second visit (2012–2014), after 4-year interval. The local institutional review board approved the study that was conducted following the ethical rules for human experimentation stated in the Declaration of Helsinki, and all participants signed an informed consent.

**Figure 1:**
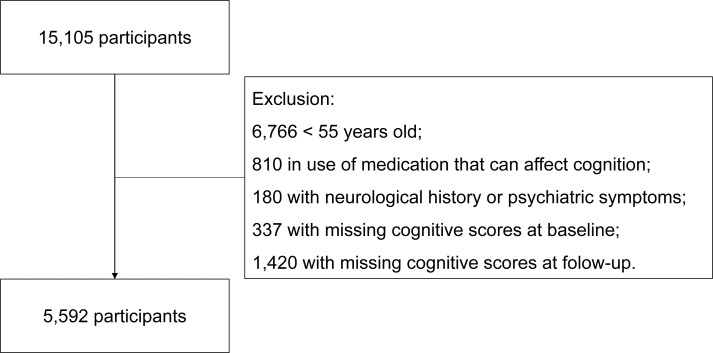
Sample selection flowchart.

For this study, participants were excluded if they reported diagnoses of neurological diseases at the baseline (e.g., stroke, concussion, brain tumor, multiple sclerosis, Parkinson's disease, dementia, and epilepsy), if they were using any medication with psychoactive effects (e.g., benzodiazepines, neuroleptics, antiparkinsonian agents, anticonvulsants, sedating antihistamines, lithium, α-adrenergic agonists, and tricyclic antidepressants), and those who had psychiatric symptoms based on mental health evaluation (Figure 1). We also excluded participants with missing cognitive test scores at baseline or follow-up evaluations. Among 7,066 eligible participants who were 55 years old at the second visit, 5,592 were considered the final sample (Figure 1).

### Neuropsychological assessment

Baseline assessment used the standardized memory tests from the Consortium to Establish a Registry for Alzheimer's Disease (CERAD)^14^ validated for the Brazilian population^15^ to assess learning, delayed word recall, and recognition (CERAD Word List Test [WLT]). The recognition score is the number of corrected classified words that belonged to the list (0–10 points) with penalization for including distractors (the number of correctly identified words minus false-positive errors — distractors words identified as part of the list). The baseline assessment also included the semantic verbal fluency (SVF) and phonemic verbal fluency (PVF) tests (animals and letter F, respectively)^16,17^ and the Trail Making Test B (TMT-B)^18^. All tests were performed using the Brazilian-Portuguese version. Follow-up assessment used the same cognitive measures, except in the case of the verbal fluency tasks. Letter A replaced the PVF of letter F, and the SVF of animals was replaced by vegetables in order to reduce learning effects. However, we used previously test equated scores^19^. Equated scores aim to guarantee that the distinct versions of the verbal fluency tests measure the construct with the same difficulty level, by transforming one test score into the same metric and range of values from another test. Trained examiners administered the tests in a fixed order during one single session, and all psychometric environment requirements were met (a quiet, lighted, and free of distractors environment)^20^.

### Statistical analysis

We evaluated the retest effects using three approaches to clarify if there is a real increase in cognitive performance, and we tested distinct possibilities to correct retest scores to be used in clinical studies. Two approaches were inspired on the study by Racine et al.^21^ The comparative approach was no retest correction, using the raw cognitive scores at follow-up. The first approach was the mean difference correction^21^. This approach first subtracts the observed baseline score from the follow-up score and then the mean of the difference of the sample is considered the retest effect. Then, the mean retest effect was subtracted from the follow-up value to obtain the mean difference corrected score for follow-up. The second approach was the predicted difference correction^21^. This regression-based approach first uses the baseline score to predict a retest score (follow-up). Then, the regression predicted retest score is subtracted from the observed score at follow-up to obtain the retest effect. Finally, the retest effect was added to the observed baseline score at baseline to obtain the predicted difference corrected score for follow-up. All assumptions required to perform the linear regression models were met. Considering that the regular method for these corrections is to use a control sample to first extract the retest effect and subsequently apply the correction to the entire sample, we used a subsample of participants that previously built a robust normative data, based on the absence of risk factors and objective cognitive decline (for the complete description, see Bertola et al.)^22^. Briefly, this robust subsample of the ELSA-Brasil was composed of 3,888 participants who, after exclusion criteria (e.g., baseline and follow-up self-reported stroke, use of psychoactive medications, missing cognitive scores, and Reliable Change Index [RCI]>-1.96), were considered not having possible cognitive decline after 4-year interval. This subsample offers the mean retest effect at the second approach, so it could be subtracted from the follow-up value for the entire sample. Similarly, this subsample provided the regression coefficients needed to predict the retest score (follow-up) for the entire sample.

We calculated the within-subject t-test to compare the baseline score with no correction, mean predicted difference correction, and predicted difference correction.

The third approach is an RCI^23^. Considering that there are distinct options to compute the RCI, we decided to use the Crawford and Howell's method once their mathematical expression corrects for practice and regression to the mean in the predicted score and individualizes error term based on the initial test score^24^. Basically, the individual's predicted retest score is subtracted from their actual retest score and then divided by a standard error (the complete formula is published and can be accessed from Hinton-Bayre)^23^. This approach extracted the correlation value, baseline and follow-up mean, standard deviation, and variance values from the same robust normative subsample. The regression coefficient to obtain the predicted score was derived using a weighted least square model to account for heteroscedasticity. This approach does not produce a corrected score, but rather indicates if the observed change in scores from baseline to the follow-up visit is a meaningful score change or a change that might be attributable to retest effect and/or the test reliability. RCI score between −1.64 and 1.64 suggests cognitive stability, score below −1.64 suggests cognitive decline, and score above 1.64 suggests cognitive improvement with a 90% confidence interval.

### Retest effects and sociodemographic characteristics

To verify if sociodemographic characteristics can distinctly affect the occurrence of retest effects, we performed linear regression analysis for each task retest effect from the predicted difference correction method. Age, education, and sex were added as predictors of the retest effect.

## RESULTS

Table 1 shows the characteristics of the sample (n=5,592). Overall, 12% of our participants had only elementary school levels (up to 10 years of schooling), 56% were white, and 55% were women. The raw mean cognitive scores on baseline and follow-up revealed a small increase after the 4-year interval (Tables 2), with exception of PVF task, revealing retest effects after within-subject t-test (Table 3). The approaches of mean difference correction and the predicted difference correction showed scores slightly lower than the baseline ones (Tables 2 and 3).

**Table 1:** Descriptive characteristics of the sample (n=5,592).

		Baseline	
		M (SD)	Min–Max
Age		58.56 (5.78)	50–75
		**n**	**%**
Age (years)			
	<65	4640	83.0
	≥65	952	17.0
Sex			
	Female	3,091	55.28
Education			
	Elementary	659	11.78
	High school	1,682	30.08
	College or more	3,251	58.14
Race			
	White	3,115	56.5
	Black	1,413	25.63
	Brown	764	13.86
	Asian	169	3.07
	Other	52	0.94

M: mean; SD: standard deviation.

**Table 2:** Mean and standard deviation for each cognitive test, considering no correction, mean difference correction, predicted difference correction, and Reliable Change Index (n=5,592).

	Baseline	Follow-up			RCI
		No correction	Mean difference correction	Predicted difference correction	
	M (SD)	M (SD)	M (SD)	M (SD)	M (SD)
WLT Learning	20.94 (3.85)	21.11 (3.98)	20.73 (3.98)	20.73 (4.95)	-0.20 (0.45)
WLT Recall	6.83 (1.97)	6.96 (2.04)	6.72 (2.03)	6.72 (2.53)	-0.27 (0.42)
WLT Recognition	9.52 (0.89)	9.62 (0.80)	9.50 (0.80)	9.50 (1.17)	0.52 (0.78)
SVF	18.25 (5.09)	18.75 (4.97)	18.10 (4.97)	18.10 (6.64)	-0.10 (0.61)
PVF	12.49 (4.40)	12.11 (4.41)	12.29 (4.41)	12.31 (5.62)	-0.00 (0.48)
TMT-B	133.56 (88.48)	129.47 (85.62)	135.33 (85.62)	134.65 (100.61)	0.48 (0.58)

RCI: Reliable Change Index; WLT Learning: Word Learning Test – Learning trial; WLT Recall: Word Learning Test – Recall Trial; WLT Recognition: Word Learning Test – Recognition Trial; SVF: semantic verbal fluency; PVF: phonemic verbal fluency; TMT-B: Trail Making Test Part B; M: mean; SD: standard deviation. Note: Higher score indicates better performance for WLT Learning, WLT Recall, WLT Recognition, SVF, and PVF, while TMT-B is measured in second, with less time indicating better performance.

**Table 3:** Within-subject t-test comparing the baseline score with follow-up no correction, mean difference correction, and predicted difference correction.

	No correction	Mean difference	Predicted difference
WLT Learning	B<F (t=-3.51, p<0.001, d=0.05)	B>F (t=4.70, p<0.001, d=0.06)	B>F (t=5.11, p<0.001, d=0.08)
WLT Recall	B<F (t=-5.35, p<0.001, d=0.07)	B>F (t=4.75, p<0.001, d=0.06)	B>F (t=5.10, p<0.001, d=0.08)
WLT Recognition	B<F (t=-7.82, p<0.001, d=0.10)	B>F (t=1.68, p<0.05, d=0.02)	B>F (t=2.63, p<0.01, d=0.03)
SVF	B < F (t=-7.22, p<0.001, d=0.09)	B>F (t=2.12, p<0.01, d=0.02)	B>F (t=2.51, p<0.01, d=0.04)
PVF	B>F (t=6.85, p<0.001, d=0.09)	B>F (t=3.55, p<0.001, d=0.05)	B>F (t=3.76, p<0.001, d=0.06)
TMT-B	B>F (t=4.09, p<0.001, d=0.05)	B<F (t=-1.76, p<0.05, d=0.02)	B<F (t=-1.20, p<0.11, d=0.02)

WLT Learning: Word Learning Test – Learning trial; WLT Recall: Word Learning Test – Recall Trial; WLT Recognition: Word Learning Test – Recognition Trial; SVF: semantic verbal fluency; PVF: phonemic verbal fluency; TMT-B: Trail Making Test Part B; B: baseline; F: follow-up.

The RCI analysis (Supplementary Table 1) suggests that the majority of the sample did not have an actual change in the cognitive performance after considering the effect for practice and regression to the mean in the predicted score and individualized error term based on the initial test score. The majority of participants (95–99%) obtained RCI scores between −1.64 and 1.64.

Education, age, and sex demonstrated to be significant predictors of retest effects for most of the cognitive scores. However, the models revealed small explained variance and small effect sizes (Table 4), indicating a minor impact of sociodemographic characteristics on the retest effects. Being older, having lower education, and being male were indicatives of marginally larger effect sizes at follow-up, but these results should be interpreted carefully. Sex was not a predictor for PVF and TMT-B.

**Table 4:** Linear regression of sociodemographic predictors of retest effect (n=5,592).

		Constant	Age	Education	Sex	Model		
						F-test	p-value	R^2^
WLT Learning	Coef.	1.40	-0.07	0.58	0.55	97.85	<0.001	0.05
	95%CI	0.48–2.32	-0.09 to −0.06	0.48–0.68	0.38–0.71			
	Eta-squared		0.02	0.02	0.01			
WLT Recall	Coef.	0.39	-0.03	0.26	0.30	8.173	<0.001	0.04
	95%CI	-0.07–0.85	-0.04 to −0.02	0.21–0.31	0.22–0.38			
	Eta-squared		0.01	0.02	0.01			
WLT Recognition	Coef.	0.09*	-0.01	0.07	0.10	34.27	<0.001	0.02
	95%CI	-0.11–0.31	-0.01–0.01	0.05–0.09	0.07–0.14			
	Eta-squared		0.00	0.01	0.00			
SVF	Coef.	-2.93	-0.05	0.85	1.67	135.75	<0.001	0.07
	95%CI	-4.19 to −1.67	-0.06 to −0.03	0.71–0.98	1.44–1.89			
	Eta-squared		0.00	0.03	0.04			
PVF	Coef.	-2.74	-0.02	1.10	0.10*	126.22	<0.001	0.06
	95%CI	-3.78 to −1.69	-0.04 to −0.01	1.00–1.22	-0.09–0.28			
	Eta-squared		0.00	0.06	0.00			
TMT-B	Coef.	-35.54	1.02	-5.58	-2.91*	23.81	<0.001	0.01
	95%CI	-55.50 to −15.60	0.72; 1.33	-7.74 to −3.42	-6.46–0.62			
	Eta-squared		0.01	0.00	0.00			

F:;

* Nonsignificant.

WLT Learning: Word Learning Test – Learning trial; WLT Recall: Word Learning Test – Recall Trial; WLT Recognition: Word Learning Test – Recognition Trial; SVF: semantic verbal fluency; PVF: phonemic verbal fluency; TMT-B: Trail Making Test Part B; 95%CI: 95% confidence interval; Coef.: coefficient.

**Figure 2:**
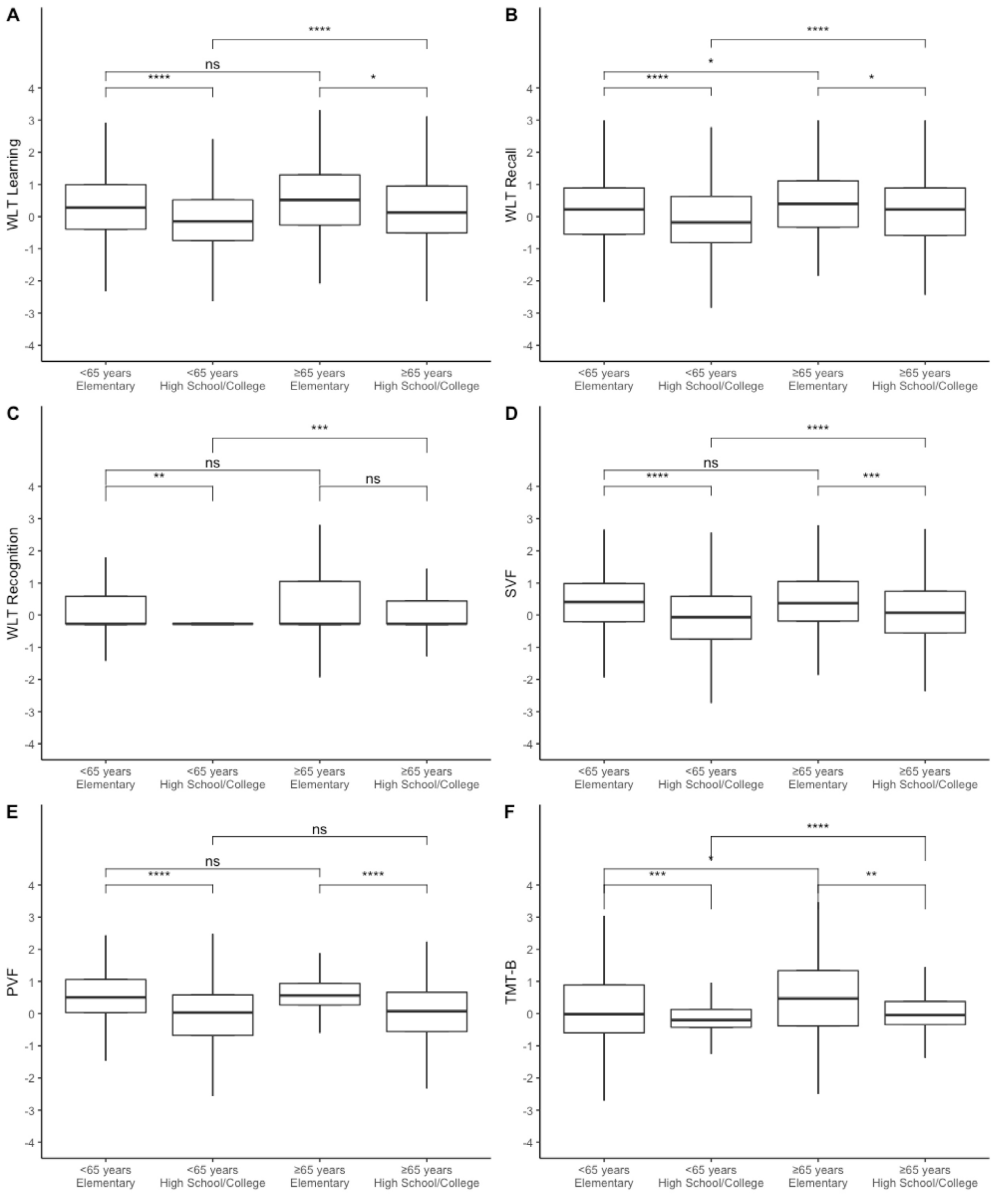
Retest effects boxplot by age (<65 years and 65 years or older) and education (elementary or high school and college or more) and groups comparisons (corrected for multiple comparisons). (A) WLT Learning: Word Learning Test – Learning trial. (B) WLT Recall: Word Learning Test – Recall Trial. (C) WLT Recognition: Word Learning Test – Recognition Trial. (D) SVF: semantic verbal fluency. (E) PVF: phonemic verbal fluency. (F) TMT-B: Trail Making Test Part B. Whiskers represent the standard deviation. ns: p>0.05, *p≤0.05, **p≤0.01, ***p≤0.001, ****p≤0.0001.

Figure 2 illustrates the retest effects as a function of age (<65 years or ≥65 years) and education group (elementary or high school [HS]+college or more), the most consistent predictors. Retest effects were more prevalent among older participants (≥65 years) with lower education (E), but younger participants (<65 years) with lower educational attainment (E) also revealed pronounced retest effects. The WLT Recognition trial (Figure 2C) was the only score with minimal or absence of retest effects and maintenance of ceiling effects, except for the participants with lower education attainment.

Considering that the educational group division resulted in uneven sample sizes, we performed additional comparisons of the retest effect among further educational groups (Supplementary Table 2). Retest effect reduced when educational attainment increase in participants younger than 65 years, except for the WLT Recognition trial. For participants aged 65 years or older, retest effect is similar to participants with elementary and HS levels, suggesting that the retest effect only reduces after a higher educational level (college or more). When educational level was kept constant and participants were compared across age, younger and older participants with elementary level did not differ in their retest effect, except for the TMT-B. Participants with HS and college levels differed, among the age groups, in WLT Learning, WLT Recall, and TMT-B.

We also performed analysis comparing the retest effect of participants with the lowest level of education (<5 years of schooling) with participants who completed the elementary school (8 years), HS (11 years), and college or more (15-16 years) (Supplementary Table 3). This additional analysis aimed to clarify the impact of the second assessment, considering that very low educated subjects underwent fewer situations of performance assessment during life. Younger participants (<65 years old) with less than 5 years of education had higher levels of retest effect only when compared with participants with HS or more (except for WLT Recognition and TMT-B). Older participants (65 years or older) with less than 5 years of education have higher levels of retest effect when compared to participants with college or more (except for WLT Recognition).

## DISCUSSION

Retest effects are common in longitudinal studies with recurrent cognitive assessments and a source of bias when not taken into account to verify cognitive change across time. We aimed to verify the occurrence of retest effects, possible approaches to correct for it, and the sociodemographic predictors of its occurrence. We found that modest retest effects occurred in the tests used at the ELSA-Brasil study (except on PVF), with some tests revealing higher effect and others revealing lower effect, especially those with the limitation of showing ceiling effects (WLT Recognition). Our results revealed smaller retest effects than usually observed in numerous studies that observed marked by improvement in test scores on the second assessment^1,2,4–8,25,26^.

Although most cited studies have a smaller follow-up interval than the ELSA-Brasil (4 years), the longitudinal increase has been reported even after a 7-year interval^27^. Additionally, a 3-year interval was associated with a mean increase of 0.30 standard deviation in scores due to retest effects^26^, a similar mean value found by our study with 4-year interval.

Our results suggest that age, education, and sex might be the potential predictors of the retest effects. However, the small effect sizes indicated that the influence of sociodemographic variables might be minimal. Gross et al.^8^ found no sociodemographic predictors in a sample of older adults, while Salthouse^4^ found that young adults revealed a higher effect. This last study compared adults aged 18–53 years with older adults aged 54–97 years that might had a true cognitive decline commonly seen in advanced ages. Middle-aged adults and young older adults might not demonstrate meaningful differences in retest effects, once age effect is not always shown. Nevertheless, we found that older adults aged 55–64 years with lower educational levels revealed higher retest effects than their more educated counterparts. Also, we found that among participants with HS or college education, adults aged 65 years or older revealed higher retest effects than their younger counterparts (aged 55–64 years).

Educational experience usually exposes the subject to recurrent schooling assessments. Higher educational levels increase the performance and knowledge about evaluation procedures, and this might contribute to less anxiety in the face of a first formal cognitive assessment. Subjects with lower education might face assessments with more anxiety symptoms for not being used to have their performance evaluated^28^. This experience might be similar to previous controlled exposures that reduce retests effects^7^.

Considering that this effect might be more prominent in lower educated subjects and that these subjects are at higher risk for presenting cognitive decline or dementia^29^, longitudinal studies from low- and middle-income countries should be extremely aware of follow-up scores correction. These subjects are a considerable proportion of older adults in these countries^30^, and higher practice effects might cover a true cognitive decline.

Once the correction of follow-up scores is needed, there are two main options to avoid biased cognitive scores: the mean difference and the predicted difference corrections. Nonetheless, considering the possible impact of sociodemographic predictors on this effect in this sample, we recommend that further studies choose the predicted difference correction. This approach allows the inclusion of relevant predictors in the regression analysis to improve the correction of retest effects for each research question asked and additionally account for the effect of regression to the mean^21,31^.

The RCI results also highlighted that the majority of the participants did not increase their cognitive performance after 4 years. Most of the small differences in scores from baseline to follow-up might be due to test reliability and practice effect susceptibility. The RCI did not revealed higher proportion of lower educated (elementary level) participants with significant decreased or increased scores on the second assessment when compared to HS and college education, except for the TMT-B (20% revealed an improvement). Stein and colleagues studied the CERAD battery and found that the RCI analysis revealed that changes in the test battery after 3 years can be interpreted with uncertainty due to possible measurement errors, practice effects, and even normal age-related cognitive decline^32^. The RCI is a limited approach that only allows for the comparison of two evaluation at a time and is not suitable for longitudinal studies with multiple cognitive assessments, in which regression approaches are more recommended^33^.

Previous to baseline or in-between waves exposure to external cognitive assessment might increase or decrease the retest effects. The absence of this information in the ELSA-Brasil questionnaire is a limitation to our comprehension of additional factors that might affect the retest effects. Given that we only have available data for two waves, we could not apply a model-based correction^21^. Further studies with this approach are recommended, including the interaction terms with time when future follow-up data become available. There are other approaches (e.g., indicator of the first cognitive visit, number of prior testing occasions, and square root of the number of prior testing occasions) to account for practice effects in the face of multiple follow-ups, and how the effects are specified can lead to considerable differences in estimated rates of cognitive change^34^.

Our study has some limitations. We do not have information if the participant has been exposed to other out-of-the study cognitive assessment previously to the baseline assessment. We could not control for other sources that might have contributed to the increase in follow-up scores. However, it is highly unlikely that participants were exposed to a cognitive assessment or rehabilitation outside the ELSA-Brasil during the study period. The absence of a test validity assessment on the battery also contributes to our limited interpretation of why the low educated participants revealed a higher practice effect. However, considering the sample selection, it is unlikely that the participants were not sufficiently engaged to perform the cognitive battery to consider the scores unreliable. Finally, the tests have reliability studies inside the ELSA-Brasil study and validity studies in other Brazilian samples, and thus the complete absence of bias cannot be guaranteed.

Our study addressed and contributed to the understanding of predictors of retest effects using a diverse socioeconomic sample. Moreover, we identified and recommended the best retest correction for an extensive data set with the potential to explore factors associated with cognitive decline in a low- to middle-income country. Future studies with the ELSA-Brasil data set will contribute to increasing the knowledge about protective and risk factors for health and pathological aging, through unbiased cognitive change scores.
